# Fiber Intake and Insulin Resistance in 6374 Adults: The Role of Abdominal Obesity

**DOI:** 10.3390/nu10020237

**Published:** 2018-02-20

**Authors:** Larry A. Tucker

**Affiliations:** Department of Exercise Sciences, Brigham Young University, Provo, UT 84602, USA; tucker@byu.edu; Tel.: +01-801-422-4927

**Keywords:** complex carbohydrate, homeostatic model assessment, obesity, waist circumference, body fat, diabetes

## Abstract

A cross-sectional design was used to evaluate the relationship between fiber intake and insulin resistance, indexed using HOMA (homeostatic model assessment), in a National Health and Nutrition Examination Study (NHANES) sample of 6374 U.S. adults. Another purpose was to test the influence of covariates on the association. A third aim was to compare HOMA levels between two groups based on the recommended intake of 14 g of fiber per 1000 kilocalories (kcal). Fiber intake was measured using a 24-h recall. With demographic variables controlled, results showed that HOMA differed across High, Moderate, and Low fiber categories (*F* = 5.4, *p* = 0.0072). Adjusting for the demographic variables, the possible misreporting of energy intake, smoking, and physical activity strengthened the relationship (*F* = 8.0, *p* = 0.0009), which remained significant after adjusting for body fat (*F* = 7.0, *p* = 0.0019) and body mass index (BMI) (*F* = 4.9, *p* = 0.0108), with the other covariates. However, the fiber–HOMA relationship was eliminated after adjusting for waist circumference (*F* = 2.3, *p* = 0.1050). Dividing participants based on the recommended 14-g standard resulted in meaningful HOMA differences (*F* = 16.4, *p* = 0.0002), and the association was not eliminated after controlling for waist circumference. Apparently, adults with high fiber consumption have less insulin resistance than their counterparts. However, much of the association is due to differences in waist circumference, unless the recommended intake of fiber is attained.

## 1. Introduction

Type 2 diabetes is a primary risk factor for cardiovascular disease, substantially increasing the risk of both coronary heart disease and stroke [1,2,3]. Moreover, all-cause mortality is 50% higher in diabetics than their counterparts following myocardial infarction [4]. Unfortunately, the prevalence of diabetes is increasing, with 11.9% of the U.S. adult population currently diseased compared to just a few decades ago when the prevalence was 8.8% [5].

Even before the onset of diabetes, elevated blood glucose levels are hazardous. When the body is unable to respond to a glucose load with the appropriate amount of insulin to mediate glucose uptake, injury tends to occur. This condition is called insulin resistance. It precedes type 2 diabetes [6,7]. In a recent study by Hulman et al., results showed that elevated 30-minute blood glucose levels during the oral glucose tolerance test in non-diabetic adults were strongly associated with the development of future diabetes and all-cause mortality [6]. Other research indicates that insulin resistance increases the risk of numerous diseases, including hypertension, coronary heart disease, stroke, and cancer [8,9,10].

A number of factors contribute to the development of insulin resistance, including smoking, physical inactivity, and obesity [11,12,13,14]. Diet also plays a significant role in the development of insulin resistance [15,16,17,18]. Investigations indicate that diets high in fat, saturated fat, and/or simple sugars often lead to insulin resistance [15,16,17,18].

Consumption of a healthy diet, particularly one with significant amounts of dietary fiber, appears to be a good strategy for minimizing a multitude of disorders, including insulin resistance, according to reviews by Anderson et al. [19] and McAuley et al. [20]. A number of studies show that the risk of insulin resistance is reduced and/or insulin sensitivity is increased when fiber is consumed abundantly [19,20,21,22,23,24,25]. However, a comparison of these investigations is difficult, since a broad array of designs and measurement methods have been employed to evaluate the fiber and insulin resistance relationship. Moreover, few investigations have controlled for key, potential confounding factors, such as body fat and abdominal obesity. Finally, to date, the relationship between fiber intake and insulin resistance has never been studied in a population-based sample generalizable to the U.S. adult population. Given the seriousness of type 2 diabetes, the significance of insulin resistance, the potential effect of fiber consumption on insulin sensitivity, and the unknown mediating influence of body fat and abdominal obesity in the fiber–insulin resistance relationship, the present National Health and Nutrition Examination Survey (NHANES) was conducted. 

The principal aim of the current investigation was to determine the extent to which 6374 women and men, 20–84 years of age, representative of non-institutionalized, civilian adults in the U.S., differ in insulin resistance across levels of fiber intake. Another objective was to ascertain the extent to which potential mediating variables, including age, gender, race, smoking, body mass index (BMI), body fat percentage, abdominal obesity, and physical activity, influence the relationship between fiber intake and insulin resistance. A third purpose was to compare insulin resistance (HOMA) levels between two groups based on the U.S. 2015–2020 Dietary Guidelines recommendation of 14 g of fiber intake per 1000 kilocalories (kcal) [26].

## 2. Materials and Methods

### 2.1. Study Design

A cross-sectional design was used to conduct the present study, and data from the National Health and Nutrition Examination Survey (NHANES) were used to answer the research questions. NHANES is a large, ongoing research program that is part of the National Center for Health Statistics and the Centers for Disease Control and Prevention. NHANES focuses on the assessment of hundreds of variables related to human nutrition and health. Data from four survey cycles covering eight consecutive years, 1999–2006, were used in the present study. These eight years were used in the present study, because they are the only years NHANES measured body fat percentage in men and women using DXA (dual energy X-ray absorptiometry). Significant details about each variable used in the present study can be accessed online [27].

Written informed consent was obtained from all participants. NHANES data collection was approved by the Institutional Review Board (IRB) of the National Center for Health Statistics, now referred to as the Ethics Review Board (ERB). The ethical approval code for NHANES data collection of 1999–2004 is #98-12 and 2005-2006 is #2005-06.

### 2.2. Sample

A total of 6374 adults, 3321 women, and 3053 men were included in the present investigation. Because NHANES employs a sophisticated, multistage probability sampling strategy to select participants, the present sample was representative of the adult population of the United States. Specifically, NHANES selects individuals using four stages: (1) Primary Sampling Units (PSUs) are ÷ selected, which includes counties or groups of small counties; (2) blocks or segments within PSUs are selected; (3) households within blocks or segments are selected; (4) individuals within households are selected.

Participants were chosen from 117 randomly selected clusters and 58 strata. For the current investigation, a subsample of individuals was asked to fast and participate in a morning lab session to have their fasting plasma glucose and fasting insulin measured (which were necessary to calculate HOMA). For the 1999–2000 survey cycle, 2986 individuals, ages 12 years and older, fasted and produced valid blood results, usable for calculating HOMA. The number of specimens was 3379 for the 2001–2002 cycle, 3120 for 2003–2004, and 3080 for 2005–2006. For the present study, age was delimited to adults, 20–84 years old. Diabetics were not included. Moreover, only women and men who had complete NHANES data, including dietary fiber and energy intake values, fasting glucose and insulin, age, gender, race, smoking, body mass index (BMI), body fat percentage, waist circumference, and physical activity level, were included in the study—a total of 6374 individuals.

The Institutional Review Board (IRB) of the National Center for Health Statistics, now referred to as the Ethics Review Board, approved the NHANES data collection and allowed the data files to be posted on their website for public use. Written informed consent was obtained by NHANES from each participant prior to data collection [28].

### 2.3. Measures

In the present investigation, insulin resistance was the outcome variable. It was indexed by the homeostatic assessment model (HOMA) and was calculated using two variables: fasting plasma glucose and fasting insulin. The exposure variable was grams of total fiber consumed per 1000 kilocalories (kcal). Eight variables, age, gender, race, smoking, body mass index (BMI), body fat percentage, waist circumference, and level of physical activity, were evaluated as potential mediating variables.

#### 2.3.1. Insulin Resistance

Insulin resistance was indexed using the following HOMA formula: fasting insulin (μU/mL) × fasting glucose (mg/dL) ÷ 405. Fasting insulin and fasting glucose data were obtained through the NHANES measurements of diabetes profiles [29,30]. Adults with diabetes (defined as having a fasting glucose of ≥126 mg/dL, being told by a physician that one is diabetic, or using insulin or an oral medication for diabetes) were not included in the study.

Subjects who were assigned to NHANES morning sessions were asked to fast for 9 h in preparation for a fasting blood draw. NHANES provides detailed information about the glucose and insulin assessment procedures [30].

#### 2.3.2. Fiber Intake

A 24-h dietary recall was administered by NHANES using a computer-assisted interview [31,32]. The dietary assessment was used to collect detailed information about all foods and beverages consumed during a 24-h period prior to the interview (midnight to midnight). Total fiber consumption was calculated by NHANES based on the recall data. Total energy consumption was also determined by NHANES, allowing fiber intake to be expressed as grams per 1000 kcal. Each dietary recall interviewer had at least 10 credits in food and nutrition courses and was a college graduate in Food and Nutrition or Home Economics. Interviewers were bilingual and interviews were administered in a private setting in the NHANES mobile examination center (MEC). Interviewers followed scripts provided in the system, and the computer-assisted program provided a standardized interview design. A multi-pass format was used during the interview. The diet recall included food probes that have been used in previous United States Department of Agriculture (USDA) and NHANES surveys. 

#### 2.3.3. Covariates

The study controlled for differences in age, gender, race, misreported energy intake, cigarette smoking, body mass index (BMI), body fat percentage, abdominal obesity, and physical activity. Age, gender, and race were considered the demographic covariates. NHANES used the following race/ethnicity categories: Non-Hispanic White, Non-Hispanic Black, Mexican American, Other Race, including Multi-Racial, and Other Hispanic. Pack years, which reflect participants’ cumulative exposure to tobacco smoke, were used to index cigarette smoking. The number of cigarettes smoked per day and the number of years the person has smoked were multiplied and then divided by 20 [33].

BMI was calculated using the formula: weight (kg) divided by height in meters squared [34]. Weight was taken using a Toledo digital scale while the subject was wearing only underwear, a disposable paper gown, and foam slippers [35]. Standing height was measured with a fixed stadiometer with a moveable headboard [35].

Body fat percentage assessed by DXA was evaluated by NHANES from 1999–2006 only. Hence, these were the years used in the present study. Because of the radiation exposure, pregnant females were not scanned. The whole body DXA scan used a Hologic QDR 4500A fan-beam densitometer (Hologic, Inc., Bedford, Massachusetts, USA) [35,36]. Participants taller than 6.42 feet or more than 300 pounds (136.36 kg) were excluded because of DXA table limitations [35,36]. Due to these exclusions for large body sizes, missing DXA data were not missing at random, leading the NHANES analysts to perform multiple imputations to complete the missing data for analysis. Details of the multiple imputation protocol are described elsewhere [36,37]. The body fat percentage variable was treated as categorical. Participants were divided into gender-specific quintiles with approximately 1150 individuals in each quintile. Those with totally missing DXA data, including those without imputation values, formed a sixth category (*n* = 633). 

Abdominal obesity was indexed using waist circumference. The measure was obtained by trained health technicians [35]. Experienced trainers, National Center for Health Statistics staff, contractor staff, and gold standard examiners regularly monitored health technician performance. The waist measurement was performed in a specially equipped room in the mobile examination center (MEC). The health technician was assisted by a trained recorder. The technician and recorder worked as a team to position, measure, and record the measurements accurately. The measuring tape was placed around the trunk in a horizontal plane just above the uppermost border of the ilium. A mirror was used to ensure horizontal alignment of the tape. The tape was to be pulled snug, but not compress the skin. The measurement was taken at the end of a normal expiration [35]. 

Physical activity level was assessed using four sentences, each describing a different level of physical activity [38]. Participants selected the sentence that best described their daily activities: (1) You sit during the day and do not walk about very much; (2) You stand or walk about during the day, but do not carry or lift things very often; (3) You lift light loads or you have to climb stairs or hills often; and (4) You do heavy work or carry heavy loads.

Misreported energy intake is a common problem when dietary information is self-reported [39]. Inaccurate reporting of energy intake could bias the fiber consumption results of this investigation, because fiber intake was expressed as grams of fiber consumed per 1000 kcal. Underreporting of energy intake would produce results that cause fiber intake values (per 1000 kcal) to appear higher than they actually are. 

In the present study, a strategy that employs self-reporting, the 24-h recall method, was used to assess dietary intake. Therefore, some misreporting likely occurred, particularly under-reporting of energy consumption. Mendez et al. discusses a variety of methods to minimize the influence of misreporting [40]. In the present study, energy intake was estimated and compared to self-reported values, and the mathematical difference between the estimated and self-reported values was used as a covariate in regression models to adjust for misreported energy intake. 

Specifically, resting metabolic rate (RMR) was estimated from weight, height, gender, and age by using the Mifflin RMR formula [41]. To estimate physical activity level (PAL), the physical activity (PA) variable discussed above was utilized. The PA variable is an ordinal, categorical variable with four levels. Each category was assigned to a PAL level, 1.45, 1.55, 1.65, and 1.75, respectively. Multiplied together, RMR and PAL were used to estimate total energy expenditure. 

As a check of concurrent validity, the relationships between estimated energy intake and BMI, waist circumference, and body fat percentage, respectively, were strong (*F* = 4013.8, *p* < 0.0001; *F* = 6181.8, *p* < 0.0001; *F* = 2210.1, *p* < 0.0001), with gender, race, and PA controlled statistically. However, there was no relationship between self-reported energy intake and BMI (*F* = 0.4, *p* < 0.5470) or waist circumference (*F* = 0.0, *p* < 0.8372), and the association with body fat percentage was inverse (*F* = 32.1, *p* < 0.0001) after adjusting for the same covariates. 

In summary, energy expenditure was estimated using Mifflin’s RMR formula and PAL. To estimate misreported energy intake, this value was subtracted from the self-reported energy intake value derived using the 24-h recall. Misreported energy intake was controlled statistically as part of the regression models reported in the Results.

### 2.4. Statistical Analysis

Findings associated with NHANES research are special, because they can be generalized to the U.S. civilian, non-institutionalized population. This is because of random sampling and the use of person-level sample weights. When unequal selection probability is applied, the sample weights yield unbiased national estimates.

Although a sample of almost 6500 individuals would be presumed to provide substantial statistical power, the multi-level, nested sampling approach of NHANES resulted in only 59 degrees of freedom (df) in the denominator, in the present study. The 59 df were calculated by subtracting the 58 strata from the 117 clusters. In the present study, for example, an *F*-ratio of 3.70 and 1 df in the numerator resulted in a *p*-value of 0.0593. 

Descriptive data, including means ± standard errors for continuous variables and frequencies for categorical variables, are reported. Each descriptive value includes adjustments based on the complex sampling design of NHANES by incorporating strata and clusters, as well as individual sample weights for the subsample of fasting participants used in the current study. Proc SurveyMeans was employed to generate weighted means that represent values for the U.S. population, and Proc SurveyFreq was used to calculate weighted frequencies, which are also generalizable to the U.S. adult population [42].

The primary outcome variable of the current study was insulin resistance, indexed using HOMA. Because the HOMA variable deviated significantly from a normal distribution, HOMA values were log-transformed prior to modeling. The exposure variable was fiber consumption, expressed as total grams of fiber consumed per 1000 kcal. The principal analysis was to determine the extent to which mean HOMA levels differed across gender-based quartiles of fiber intake using linear regression and the Proc SurveyReg procedure. The two middle-quartiles were collapsed forming three categories of fiber consumption: Low (25%), Moderate (50%), and High (25%). For women, cut-points for 24-h fiber intake (grams per 1000 kcal) were: Low (≤5.0), Moderate (5.1–9.8), and High (>9.8). For men, cut-points (grams per 1000 kcal) were: Low (≤4.2), Moderate (4.3–8.5), and High (>8.5). Because labels such as Low, Moderate, and High can be misinterpreted, quartile 1 (Q1), quartiles 2 and 3 (Q2-Q3), and quartile 4 (Q4) were conjoined with each label to emphasize that sex-specific quartiles were used to define the fiber intake categories. To test the extent to which the association between fiber intake and insulin resistance was influenced by the covariates (age, gender, race, smoking pack-years, physical activity, BMI, waist circumference, body fat percentage, and misreported energy intake), these factors were controlled statistically using partial correlation. Adjusted means were calculated using the least-squares means procedure [43].

In another analysis, participants were grouped according to the fiber intake recommendation (14 g per 1000 kcal) of the U.S. Dietary Guidelines for Americans, 2015–2020 [26]. Participants with less than 14 g of fiber intake per 1000 kcal per day (those not reaching the Guidelines) were compared to those consuming 14 g or more (those meeting the Guidelines). 

All *p*-values were two-sided and statistical significance was accepted when alpha was <0.05. The statistical analyses were performed using SAS, Version 9.4 (SAS Institute, Inc., Cary, NC, USA) [43].

## 3. Results

To produce findings that are generalizable to the adult population of the United States, individual NHANES sample weights were employed in the present study. Mean age (±SE) of the sample was 44.1 ± 0.4 years, average fiber intake was 15.7 ± 0.2 g per day, and mean fiber intake per 1000 kcal was 7.3 ± 0.1 g per day. A total of 93.6% ± 0.4 of the participants failed to reach the U.S. Dietary Guidelines recommendation of 14 g of fiber per 1000 kcal per day. When the self-reported energy intake values were replaced with the estimated values, mean fiber intake per 1000 kcal was 6.6 ± 0.1 g per day, and 95.2% ± 0.4 failed to reach the recommended 14 g of fiber per 1000 kcal per day. Mean HOMA for all participants was 2.2 ± 0.05. Table 1 shows additional descriptive information for the sample. 

With grams of fiber intake per 1000 kcal and HOMA both treated as continuous variables, there was a significant inverse, linear relationship (*F* = 7.7, *p* = 0.0075), with 1 and 59 degrees of freedom. There was no curvilinear association. Specifically, for each 10 g decrease in fiber (per 1000 kcal), HOMA was 0.4 points higher, on average. After adjusting for differences in the demographic variables (age, gender, and race), the association was strengthened (*F* = 10.2, *p* = 0.0022). With differences in the demographic variables, plus smoking, PA, and misreported energy intake controlled, the relationship was further strengthened (*F* = 19.0, *p* < 0.0001). Additionally, when estimated energy intake was substituted for self-reported energy intake to calculate fiber intake per 1000 kcal, the association between fiber and HOMA was much stronger (*F* = 79.8, *p* < 0.0001), with the demographic variables controlled. Specifically, for each 10 g decrease in fiber (per estimated 1000 kcal), HOMA was 0.7 points higher, on average. However, when estimated energy intake was used in place of self-reported energy intake, and age, gender, race, smoking, physical activity, and waist circumference were controlled, the relationship was no longer significant (*F* = 2.98, *p* = 0.0895).

Table 2 shows the extent to which mean HOMA levels differed across the three categories of fiber intake per 1000 kcal: Low (Q1), Moderate (Q2-Q3), and High (Q4). In each analysis, there were 59 df in the denominator. After adjusting for differences in the demographic variables, HOMA differed significantly across the three levels of fiber intake (*F* = 5.4, *p* = 0.0072). As displayed in Table 2, adults in the High (Q4) fiber category had significantly lower HOMA levels compared to those in the Low (Q1) and Moderate (Q2-Q3) groups. There was no difference between the Low and Moderate categories. 

Adjusting for differences in misreported energy intake (MEI), along with the demographic covariates, strengthened the relationship between fiber and HOMA (*F* = 9.9, *p* = 0.0002). Including smoking pack-years and PA level with the demographic covariates and MEI increased the HOMA differences across the fiber intake categories (*F* = 13.9, *p* < 0.0001). Including DXA-measured body fat percentage with the above covariates weakened the relationship and reduced the magnitude of the mean differences in HOMA (*F* = 7.0, *p* = 0.0019), as shown in Table 2. Substituting BMI in place of body fat percentage, along with the other covariates, resulted in a weaker relationship (*F* = 4.9, *p* = 0.0108), but it remained statistically significant. However, substituting abdominal obesity (waist circumference) for BMI, along with the other covariates (i.e., age, gender, race, MEI, pack-years, PA), weakened the relationship between fiber intake (per 1000 kcal) and HOMA so that it was no longer significant (*F* = 2.3, *p* = 0.1050).

Because of the powerful mediating effect of abdominal obesity (waist circumference) on the relationship between fiber intake and insulin resistance, the 3-way interaction between abdominal obesity and insulin resistance and also fiber intake and abdominal obesity was evaluated. Post-hoc analysis using sample weights, strata, and clusters, so the results can be generalized, revealed a strong correlation between waist circumference and HOMA (*F* = 1371.8, *p* < 0.0001). Differences in waist circumferences alone accounted for 35% of the variance in HOMA. The analysis also showed that fiber intake (grams per 1000 kcal) and abdominal obesity were inversely related (*F* = 9.3, *p* = 0.0034). As fiber intake (grams per 1000 kcal) increased, waist circumferences tended to be smaller. In short, there was 3-way correlation between fiber intake, abdominal obesity, and insulin resistance in U.S. adults. However, results also indicated that abdominal obesity plays a much bigger role in insulin resistance than fiber intake, although both are significant contributors. 

With subjects categorized based on whether or not they met the U.S. Dietary Guidelines recommendation of 14 g of fiber per 1000 kcal per day, results showed that adults who met or exceeded the guidelines (*n* = 501) had HOMA levels that were 34.7% lower than those who did not consume at least 14 g of fiber per 1000 kcal (*n* = 5873), after controlling for differences in the demographic variables, MEI, smoking, and physical activity (*F* = 16.4, *p* = 0.0002). In contrast to the quartile-based findings, mean differences in HOMA were weakened, but remained significant after adjusting for differences in age, gender, race, MEI, smoking pack-years, physical activity, and waist circumference (*F* = 4.5, *p* = 0.0390). 

## 4. Discussion

The primary purpose of the present study was to determine the relationship between dietary fiber intake (grams per 1000 kcal) and insulin resistance (HOMA) in a randomly selected sample of 6374 adults, ages 20–84 years, representative of the U.S. population. Another objective was to ascertain the extent to which the association between fiber consumption and insulin resistance was a function of a number of potential confounders, including multiple measures of obesity and body composition. A third aim was to determine the extent to which HOMA levels differed across groups based on fiber intake above and below the recommended level of 14 g per 1000 kcal. 

Results indicated that fiber intake (grams per 1000 kcal) was linearly and inversely related to insulin resistance with both measures treated as continuous variables—as fiber intake increased, insulin resistance tended to decrease. Treating fiber intake as a categorical variable, comparing the lowest and highest quartiles of fiber consumption showed that adults with Low (Q1) fiber intake had HOMA levels that were 19% higher than those with High (Q4) fiber intake (*F* = 5.4, *p* = 0.0072). Additionally, the relationship between fiber intake (per 1000 kcal) and HOMA remained significant after adjusting for a number of covariates. However, when abdominal obesity (waist circumference) was controlled, individually or with other covariates, the fiber and insulin resistance association was eliminated. Evidently, in the present U.S. sample, if all adults had the same waist circumference, there would not be a significant relationship between fiber intake and insulin resistance. 

Using a variety of designs and measurement methods, other investigations have studied the association between fiber intake and insulin resistance. Focusing on baseline data from the Inter99 study, Lau et al. [22] used Danish men and women to investigate the relationship between fiber intake and HOMA. A self-administered food frequency questionnaire was employed to measure fiber consumption. Fiber intake was expressed as total grams, not grams per 1000 kcal. The cross-sectional association between grams of fiber consumed per day and HOMA was inverse and significant, even after adjusting for potential confounders, including waist circumference. 

In the Insulin Resistance Atherosclerosis Study, Liese et al. [25] examined cross-sectional results. Consumption of fiber was assessed using an interviewer administered food frequency questionnaire. Fiber intake was not expressed per 1000 kcal. Fiber consumption was directly associated with insulin sensitivity, and inversely with insulin levels and a measure of acute insulin response. However, statistical adjustments were not made for differences in BMI or waist circumference. 

In the MESA (Multi-Ethnic Study of Atherosclerosis) study, Lutsey et al. [23] cross-sectionally investigated the relationship between whole grain intake and insulin resistance via HOMA. The researchers found that as whole grain consumption increased, HOMA levels decreased. Although BMI was measured, adjustments were not made to determine if the relationship between whole grain intake and insulin resistance was partly due to differences in BMI. 

Focusing on 264 middle-age women, Breneman and Tucker [24] studied the fiber and insulin resistance (HOMA) connection cross-sectionally. To measure fiber intake, 7-day, weighed food records were analyzed. Controlling for differences in body fat percentage weakened the relationship between total fiber and HOMA, and nullified the association between insoluble fiber and HOMA. Abdominal obesity was not measured in the study, however.

Overall, cross-sectional studies indicate that insulin resistance is inversely related to fiber consumption. However, few investigations have controlled for differences in BMI or body fat, and fewer have adjusted for abdominal obesity. Therefore, the degree to which the association between fiber intake and insulin resistance is a function of differences in abdominal obesity remains unclear. 

Some, but not all, experimental studies have shown a significant relationship between fiber intake and reduced insulin resistance and/or improved glycemic control. Most have used soluble fiber as the treatment. For example, Li et al. [44] directed a double-blind, randomized controlled trial with a twice-daily dose of supplemental soluble fiber for 12 weeks. Compared to controls, the treatment group experienced a significant reduction in insulin resistance. Similarly, in a double-blind, placebo-controlled cross-over study, Landin et al. [45] tested the effect of guar gum, a soluble fiber, administered three times per day for 6 weeks with 2-week run-in and wash-out periods in 25 men. Compared to the placebo, the guar gum treatment decreased fasting blood glucose levels, but fasting insulin was unchanged. Likewise, Aro et al. [46] tested the effect of guar gum fiber administered three times per day with the main meals for three months using a double-blind, cross-over design with nine participants. Results showed that basal and post-prandial hyperglycemia were reduced significantly. However, insulin levels were not influenced. Lastly, Vuksan et al. [47] treated 11 adults with either soluble fiber-enriched biscuits or control biscuits for two 3-week treatment periods with a 2-week washout period using a cross-over design. Fasting glucose and insulin levels were not influenced by the treatment.

Insoluble fiber has also been shown to reduce insulin resistance. Robertson et al. [48] utilized a 4-week supplementation period and a cross-over design to evaluate the effects of resistant starch on insulin sensitivity, which was assessed by HOMA and the euglycemic-clamp. There was no effect on β-cell function or fasting insulin sensitivity when measured by HOMA, but the clamp method showed that the resistant starch improved insulin sensitivity significantly. Favorable results were also identified in adults with Metabolic Syndrome over 8 weeks using insoluble fiber and a placebo-controlled investigation [49]. 

Overall, experimental studies have shown mixed findings. Fasting and/or post-prandial glucose levels have generally been reduced by fiber treatments, but insulin levels have not. 

Given the design of the present investigation, the sequence and timing of the fiber, abdominal obesity, and insulin resistance relationships cannot be determined. However, logic and the literature dictate that fiber intake can influence abdominal obesity and abdominal obesity can contribute to insulin resistance [50,51,52,53,54].

Few investigations have focused on the relation between fiber intake and abdominal obesity; however, Slama et al. identified a strong inverse association between fiber intake and waist circumference in 260 Tunisian women [50]. Additionally, in a prospective cohort study with more than 89,000 European participants followed for 6.5 years, Du et al. showed that higher intakes of fiber are predictive of significant decreases in waist circumference over time [51]. 

Regarding abdominal obesity and insulin resistance, Vasques et al. determined that waist circumference is a strong predictor of HOMA in a Brazilian population [52]. Likewise, Cheng et al. showed that waist circumference is related significantly to HOMA in middle-aged and elderly Taiwanese [53]. Furthermore, in a large sample of children, adolescents, and adults, abdominal obesity, measured by waist circumference, was predictive of insulin resistance, indexed using HOMA [54].

From the literature, it appears that high fiber consumption contributes to lower risk of abdominal obesity, and higher levels of abdominal obesity contribute to higher levels of insulin resistance. Findings of the present study revealed a significant inverse relationship between fiber intake and insulin resistance, but that association was eliminated when adjustments were made for differences in waist circumference. Hence, the present investigation supports the concept that the relationship between fiber intake and insulin resistance may be due, at least in part, to differences in abdominal obesity. 

In the present study, High fiber consumption was defined as the highest gender-specific quartile. High (Q4) fiber intake was >9.8 g per 1000 kcal for women and >8.5 g per 1000 kcal for men, both far short of the recommended intake of 14 g per 1000 kcal. Yet, insulin resistance was significantly lower in men and women in the High (Q4) fiber category compared to those in the Low (Q1) or Moderate (Q2-Q3) categories. Apparently, consuming the recommended 14 g of fiber per 1000 kcal is not essential for adults desiring lower levels of insulin resistance. However, and of key importance, when participants met or exceeded the U.S. Dietary Guidelines recommendation of 14 g per 1000 kcal [26], HOMA differences could no longer be explained by variation in abdominal obesity (waist circumference). In other words, the relationship between fiber consumption and insulin resistance remained significant after adjusting for differences in abdominal obesity (waist circumference) when those meeting the 14-g fiber recommendation were compared to their counterparts. 

Multiple general mechanisms have been proposed for the inverse association between fiber intake and insulin resistance found in the present study. First, fiber intake, particularly soluble fiber, becomes gel-like in the stomach after ingestion. This slows the digestion and absorption of carbohydrates, which moderates the release of glucose, resulting in a lower insulin response [19,55]. Additionally, dietary fiber is only available in plant foods. Hence, it is possible that other plant components, besides fiber, could be responsible for the favorable link between fiber consumption and insulin sensitivity. For example, magnesium could be a mediating factor [19]. Finally, as found in the present study, it appears that differences in abdominal obesity account for a significant part of the fiber–HOMA association. Fiber-rich foods increase feelings of fullness and help curb energy intake, which tends to reduce weight gain and obesity over time [56,57]. 

A number of biological mechanisms have also been proposed to explain the fiber–insulin resistance relationship. Research indicates that fiber intake likely influences insulin sensitivity via alterations in fatty acid flux [48]. This idea is not new [58,59,60]. In a short-term study [61], high doses of insoluble fiber increased postprandial insulin sensitivity with decreased circulating levels of short-chain and nonesterified fatty acids. Additionally, in a 4-week investigation, Robertson et al. found that insoluble fiber increased ghrelin levels significantly [48], and a number of investigations have shown that ghrelin is connected with decreased insulin resistance [62,63,64]. Hence, it appears that fiber intake increases insulin sensitivity by changing both adipose tissue and skeletal muscle metabolism. This is likely a result of increases in levels of short chain fatty acids and ghrelin [48]. 

In another study focusing on mechanisms underlying the fiber–insulin resistance relationship using a randomized placebo-controlled design, insoluble fiber taken over eight weeks resulted in enhanced glucose uptake into the forearm muscle, while adipose tissue was also influenced, resulting in fatty acid suppression [49]. Additionally, fiber consumption increased gene expression for lipoprotein lipase, perilipin, adipose tissue triglyceride lipase, and hormone sensitive lipase. Fiber intake had no effect on insulin sensitivity of hepatic glucose production or plasma lipids [49]. 

Because at least part of the association between fiber consumption and insulin resistance in the present study was a function of differences in abdominal obesity, other mechanisms must be considered [65,66,67]. In a classic study conducted by Barzilai et al. [68], obese rats had visceral fat surgically removed, or a sham operation was performed. Insulin resistance was drastically reduced in the rats that had their visceral fat reduced surgically, reinforcing the role of abdominal fat in insulin resistance [68].

Abdominal fat, particularly visceral adipose tissue, releases a large quantity of free fatty acids into the portal vein that are transported to the liver. Excess abdominal fat is linked directly to the accumulation of excess liver lipids, which result in autonomous impairment of insulin signaling. As a consequence, insulin sensitivity is compromised [66]. A significant player in this process could be interleukin-6 [69]. 

Another mechanism stems from the inflammatory characteristics of abdominal fat. Visceral adipose tissues are susceptible to inflammation and accumulate macrophages that produce inflammatory cytokines, which lead to deficient insulin signaling [65]. Treatment with an anti-inflammatory agent helps to control human diabetes, highlighting the concept that inflammation is a meaningful player in insulin resistance [70].

Another possibility is that high concentrations of visceral adipose tissue serve as a marker of ectopic lipid accumulation and lipotoxicity, which occur similarly in muscle and liver tissues, resulting in insulin resistance in these tissues. 

Lastly, research shows that dietary fiber has a powerful effect on the microbiota of the human gut. As summarized by Sawicki et al. [71], fiber enriches the gut microbiota resulting in a host of favorable metabolic outcomes, including modification of short-chain fatty acid concentrations and improved insulin sensitivity. In a recent investigation by Zou et al. [72], fiber-mediated nourishment of gut microbiota was shown to protect mice against diet-induced metabolic syndrome and obesity. However, the study showed that effects were due to interleukin-22 production, but were not short chain fatty acid dependent [72]. 

The present study had multiple limitations. First, the NHANES data were cross-sectional. Therefore, causal conclusions cannot be applied. Second, dietary fiber consumption was assessed using a 24-h recall. Although this strategy has provided valuable dietary results for scores of NHANES and other epidemiologic investigations, data about fiber intake collected with greater frequency would have likely produced less measurement error and a stronger relationship between fiber intake and insulin resistance. Third, the dietary data was self-reported. Consequently, some adults tend to misreport what they actually consume. Fourth, NHANES provided data about total fiber intake, but not soluble and insoluble consumption. Details regarding consumption of soluble and insoluble fiber would have provided useful information that would have benefited the present study. Lastly, adults reporting high levels of fiber consumption could represent unique individuals with a lifestyle that is different from others. A number of variables were controlled statistically in order to minimize this potential threat, but there may be other unknown factors that could explain the relationship between fiber intake and insulin resistance. 

The present investigation also had multiple strengths. First, the NHANES data were collected by highly trained individuals using valid and well-established methods. None of the NHANES scientists were associated with the present study, so there were no internal biases. Second, many potential mediating factors were controlled statistically. Hence, the results appear to be independent of differences in age, gender, race, smoking, physical activity, BMI, and body fat percentage. However, the covariate analysis showed that much of the relationship between fiber consumption and insulin resistance was a function of differences in abdominal obesity, particularly waist circumference. Viewed separately, the relationship between waist circumference and insulin resistance was much stronger than the association between fiber intake and insulin resistance. Third, the sample employed in the present study was randomly selected by NHANES using a multistage, probability sampling design. It was large, multi-racial, and representative of the non-institutionalized, civilian population of the United States, 20–84 years of age, permitting extensive generalization of the findings. 

## 5. Conclusions

U.S. adults with high (Q4) fiber consumption were found to have significantly lower levels of insulin resistance (HOMA) than their counterparts (Q1-Q3). After controlling for differences in a number of demographic and lifestyle factors, and possible misreporting of energy intake, the inverse relationship between fiber intake and insulin resistance persisted. Nevertheless, after adjusting for differences in abdominal obesity, there was no longer a relationship between fiber consumption and insulin resistance. However, when participants were divided into two groups based on whether or not they met the recommended fiber intake standard of 14 g per 1000 kcal, insulin resistance differences were substantial, and adjusting for differences in abdominal obesity no longer eliminated the relationship. Findings of the present investigation highlight the role of abdominal obesity in the association between fiber intake and insulin resistance, and the value of consuming at least 14 g of fiber per 1000 kcal per day. 

## Figures and Tables

**Table 1:** Descriptive characteristics of the key variables (*n* = 6374).

			Percentile				
Variable	Mean	SE	10th	25th	50th	75th	90th
Fiber (g)	15.8	0.20	6.0	9.2	14.0	19.9	27.6
Men only	17.6	0.24	6.7	10.4	15.4	22.4	30.6
Women only	14.1	0.21	5.5	8.4	12.7	17.8	24.1
Fiber (g/1000 kcal)	7.3	0.08	3.3	4.6	6.5	9.2	12.3
Men only	6.8	0.08	3.1	4.2	6.0	8.5	11.5
Women only	7.8	0.11	3.6	5.0	6.9	9.8	13.2
Fiber (g/est 1000 kcal)	6.6	0.09	2.5	3.8	5.8	8.5	11.7
Men only	6.4	0.09	2.4	3.7	5.6	8.2	11.4
Women only	6.8	0.11	2.6	3.9	6.0	8.8	11.9
HOMA	2.2	0.05	0.6	0.9	1.6	2.8	4.5
Men only	2.5	0.07	0.6	1.0	1.7	3.0	4.8
Women only	2.0	0.05	0.5	0.9	1.5	2.5	4.3
Glucose (mg/dL)	95.1	0.25	83.6	88.5	94.4	98.3	107.7
Men only	97.2	0.27	86.0	91.0	96.6	102.8	109.2
Women only	93.1	0.28	82.2	86.5	92.1	98.3	105.4
Insulin (μU/mL)	9.3	0.19	2.6	4.2	6.8	11.6	18.5
Men only	10.0	0.26	2.7	4.4	7.3	12.4	19.1
Women only	8.6	0.19	2.4	4.0	6.5	10.7	17.5
Age (years)	44.1	0.39	23.6	31.0	42.2	54.0	66.9
Men only	43.4	0.41	23.1	30.2	41.3	53.3	65.6
Women only	44.8	0.44	24.0	31.7	42.9	54.8	67.9
BMI (kg/m^2^)	27.8	0.11	21.1	23.5	26.8	30.9	35.8
Men only	27.8	0.12	21.7	24.2	27.1	30.4	34.4
Women only	27.9	0.16	20.6	22.9	26.5	31.5	37.2
Body fat (%)	34.2	0.15	22.3	27.4	33.9	41.4	46.6
Men only	27.8	0.11	19.0	23.6	27.9	32.2	36.1
Women only	40.5	0.18	31.1	35.7	41.0	45.6	49.1
Waist circ. (cm)	95.5	0.28	76.4	84.4	94.2	105.0	115.4
Men only	99.0	0.35	81.2	89.2	97.6	107.4	117.3
Women only	92.3	0.39	74.1	80.7	89.9	102.1	113.1
Smoking (pack-years)	0.8	0.11	0.0	0.0	0.0	0.0	0.0
Men only	0.8	0.11	0.0	0.0	0.0	0.0	0.0
Women only	0.7	0.14	0.0	0.0	0.0	0.0	0.0
Energy intake (kcal)	2276.7	15.09	1162	1571	2116	2819	3584
Men only	2690.8	25.58	1464	1939	2558	3262	4042
Women only	1891.2	13.5	1027	1378	1795	2297	2859
Est. energy intake (kcal)	2446	7.7	1832	2059	2418	2791	3094
Men only	2790	8.3	2326	2539	2768	3012	3257
Women only	2125.8	8.7	1711	1889	2088	2327	2576

For each variable, the first row provides descriptive results for all subjects combined, for both men and women. The row labeled “Men only” includes results for only men (*n* = 3053). The row labeled “Women only” includes findings for only women (*n* = 3321). SE: standard error. Waist circ.: waist circumference. Est. energy intake: estimated energy intake (calculated using Mifflin’s resting metabolic rate (RMR) formula and physical activity level (PAL)). Gender, race, and physical activity level were categorical variables. There were 3321 women and 3053 men in the sample. There were 2555 non-hispanic whites, 832 non-hispanic blacks, 1117 Mexican Americans, 1666 other races (including multi-racial), and 204 other Hispanic. For physical activity (PA), 1358 reported minimal PA, 3432 reported light PA, 1087 reported moderate PA, and 497 reported high PA.

**Table 2:** Differences in mean HOMA values across levels of fiber intake in 6374 U.S. adults, after adjusting for covariates.

	Fiber Intake (grams per 1000 kcal)				
Variable Controlled	Low Mean ± SE	Moderate Mean ± SE	High Mean ± SE	*F*	*p*
Demographics *	2.62 ^a^ ± 0.10	2.37 ^a^ ± 0.07	2.21^b^ ± 0.07	5.4	0.0072
Demographics, MEI, pack-years, PA	2.59 ^a^ ± 0.10	2.32 ^a^ ± 0.07	2.06 ^b^ ± 0.07	8.0	0.0009
Demographics, MEI, pack-years, PA, BF%	2.55 ^a^ ± 0.10	2.28 ^a^ ± 0.07	2.14 ^b^ ± 0.07	7.0	0.0019
Demographics, MEI, pack-years, PA, BMI	2.48 ^a^ ± 0.09	2.27 ^a^ ± 0.06	2.19 ^b^ ± 0.06	4.9	0.0108
Demographics, MEI, pack-years, PA, waist	2.55 ± 0.09	2.35 ± 0.06	2.31 ± 0.06	2.3	0.1050

* Demographic variables were age, gender, and race. MEI: misreported energy intake; PA: Physical activity; BF%: body fat percentage, measured by DXA, Waist: waist circumference. ^a,b,c^ Means on the same row with the same superscript letter do not differ significantly. Low fiber intake represents the lowest gender-specific quartile. Moderate intake reflects the middle two gender-specific quartiles, and High fiber intake represents the highest gender-specific quartile of fiber intake per 1000 kcal. For women, cut-points for 24-h fiber intake (grams per 1000 kcal) were: Low (≤5.0), Moderate (5.1–9.8), and High (>9.8). For men, cut-points (grams per 1000 kcal) were: Low (≤4.2), Moderate (4.3–8.5), and High (>8.5). HOMA means have been adjusted based on the covariates in the first column.
